# Antibacterial characteristics of black currant fruit extracts

**DOI:** 10.1186/s12906-026-05535-8

**Published:** 2026-08-18

**Authors:** Stefan Kranz, Markus Heyder, Franziska Latt, André Guellmar, Bernd Sigusch, Markus Reise

**Affiliations:** Department of Conservative Dentistry and Periodontology, University Hospital, Jena, Germany

**Keywords:** Antimicrobial, Periodontitis, Chlorhexidine, Secondary plant metabolites, Caries, Dental plaque control

## Abstract

**Background:**

Our group previously reported on the high bactericidal potential of berry juices, particularly those derived from blackcurrant fruits. In the present study, we tested the efficiency of various juice fractions from blackcurrant fruits (all-fruit juice: AFJ, squeezed juice: SJ, non-dialyzable juice material: NDFM, alcoholic fraction from berry skins: AFBS, aqueous fraction from berry skins: AQFBS) in their efficiency to suppress various oral gram-positive and gram-negative bacteria.

**Methods:**

AFJ, SJ, NDFM, AFBS, and AQFBS were prepared from freshly harvested blackcurrant fruits. These fractions were used for direct contact tests with planktonic cultures of *Fusobacterium nucleatum* (F.n.), *Aggregatibacter actinomycetemcomitans* (A.a.), *Porphyromonas gingivalis* (P.g.), *Streptococcus gordonii* (S.g.), *Streptococcus mutans* (S.m.), *Streptococcus sobrinus* (S.s.), *Actinomyces naeslundii* (A.n.), and *Enterococcus faecalis* (E.f.).

**Results:**

AFJ was the most efficient fraction, causing complete suppression of F.n., P.g., and S.g., and inducing a major reduction in A.a., A.n., and E.f. colony count. Bactericidal efficiency of the juices presents as follows: AFJ> AFBS > SJ> AQFBS> NDFM. Gram-negative bacteria were more susceptible to blackcurrant extracts compared to gram-positive species. AFJ efficiency was similar to 0.2% chlorhexidine solution in suppressing F. n., P. g., and S. g.

**Conclusions:**

All-fruit juice from blackcurrant shows a strong bactericidal activity on F.n., A.a., P.g., S.g., and A.n. Further research should investigate the potential of blackcurrant fruit extracts in controlling oral biofilm formation.

## Background

 Some plant-derived foods are particularly rich in polyphenols, which belong to the group of secondary plant metabolites and can be classified into flavonoids and non-flavonoids [1]. These metabolic compounds contribute to aroma, color, astringency, and protection of plants against predators and environmental threats. Interestingly, some of these molecules also have pronounced effects on the human body and are therefore considered important phytochemicals [2–5].

In this regard, our group recently showed that especially all-fruit juices derived from various berry fruits have strong antibacterial properties. In particular, juices from blackcurrant (*Ribes nigrum*) were the most efficient in inhibiting the growth of various oral bacteria associated with periodontitis, caries and root canal infections [6].

Fruits are among the most preferred food sources with positive effects on individual health [7–12]. Because of their vitamin- and mineral-rich content, berry fruits are important components of a healthy diet. Due to high amounts of antioxidants, berry fruits have pronounced anti-inflammatory effects on several oxidative stress-related diseases [13–15].

Besides their antioxidant activity, berry polyphenols also show strong antibacterial and anti-fouling characteristics, which makes them attractive candidates for controlling food spoilage and microbial biofilm formation [16, 17].

In this regard, various authors have found that especially blackcurrant fruits are rich in flavonols (kaempferol, quercetin, and myricetin), glycosides (kaempferol-3-*O*-glycoside, quercetin-3-*O*-glycoside, and quercetin-3-*O*-rutinoside), anthocyanins, and tannins. Blackcurrant anthocyanins (delphinidin-3-*O*-rutinoside and cyanidin-3-*O*-rutinoside) are recognized as significant antimicrobial metabolites, constituting between 60% and 85% of the total phenolic content [18–20]. Due to their richness in natural bioactive compounds, blackcurrant fruits in particular show distinct health-promoting effects [20–22].

Considering the antibacterial activity of plant polyphenols, several mechanisms are already known. These include interactions with proteins and other cell wall components causing changes in their permeability and stability. Furthermore, polyphenols are able to inhibit energy metabolism, nucleic acid synthesis, and DNA repair. Polyphenols can also bind metals such as Cu^2+^, forming complexes that modify DNA stability. Overall, the inhibitory mechanisms are diverse and depend on the structure of the polyphenols, as well as the bacterial species involved [23].

In view of increasing microbial drug resistance, there is an urgent need for new and efficient alternative treatment methods.

As recently shown by our group, especially blackcurrant all-fruit juices are highly bactericidal towards many diverse oral pathogenic bacteria [6]. However, information is still lacking regarding the activity of various fruit fractions that can be obtained from blackcurrants. The present study, therefore, focuses on investigating the antibacterial activity of blackcurrant fruit fractions on diverse gram-positive and gram-negative oral bacteria. Besides the bactericidal effect of all-fruit juices, the antibacterial efficiency of alcoholic and aqueous extracts from berry skins, squeezed fruit juices, and juices from non-dialyzable fruit material was investigated as well. It was hypothesized that between these single extracts there are no significant differences in regard to their bactericidal activity (H0).

## Methods

For this study, blackcurrants (*Ribes nigrum*) grown regionally in the federal state of Thuringia, Germany, were used. The berries were collected on private land with permission from the owner. After harvesting, the fruits were washed and immediately frozen. Deep-frozen fruits (−20 °C) were transferred to the laboratory for further processing. Test solutions were prepared from berry fruits following five different protocols, and their antibacterial activity was investigated using a direct contact test. The applied protocols were adopted and modified from [24–30].

### All-fruit juice (AFJ)

Blackcurrant berries were processed using a conventional blender (Model TB6-1-6ST, AEG, Nuremberg, Germany). The resulting mixture was centrifuged at 4800 rpm for 10 min. The supernatant was collected and centrifuged again at 13.000 rpm for 5 min (Sorvall ST4 Plus, Thermo Fisher Scientific Inc., Waltham, USA) in order to remove all solid components. The resulting juice was then sterilized at 70 °C for 30 min and stored in 1.5 ml Eppendorf tubes at −20 °C.

### Squeezed berry juice (SJ)

By carefully squeezing the berries through a standard kitchen sieve (WMF, Geislingen/Steige, Germany), solids and juice were separated. The resulting juice was centrifuged at 13.000 rpm for 5 min, and the collected supernatant was sterilized at 70 °C for 30 min. Aliquots were then stored in 1.5 ml Eppendorf tubes at −20 °C.

### Non-dialyzable fruit material (NDFM)

Squeezed juice was transferred to a dialysis tube (pore size MWC = 12.00–14.00 MW) (Sigma-Aldrich GmbH, Taufkirchen, Germany) and dialyzed for 7 days at 4 °C in distilled water with constant stirring and a daily water change. The process was terminated when no visible discoloration of the distilled water was observed anymore. The content of the dialysis tube was then transferred to a sterile container, lyophilized, and stored at room temperature until use. For experimental purposes, the lyophilizate was then dissolved in PBS, according to the initially applied juice volume.

### Alcoholic fraction from berry skins (AFBS)

Berry skins were mixed with ethanol in a ratio of 1:3 and placed on a tumbling table at room temperature for 30 min. The aliquots were centrifuged at 4 °C, 1500 rpm for 5 min. The supernatants were collected and transferred to an open, sterile reaction vessel and placed alternately in an incubator at 37 °C, followed by an evaporation step at room temperature. The cycle was terminated when the whole ethanol evaporated from the solution. The resulting residues were then dissolved in dimethyl sulfoxide (DMSO, Sigma-Aldrich GmbH, Taufkirchen, Germany), and aliquots were frozen at −20 °C. For experimental purposes, the DMSO samples were dissolved in PBS up to the initial volume of the applied berry skins.

### Aqueous fraction from berry skins (AQFBS)

Berry skins were mixed with distilled water in a ratio of 1:3 and incubated on a tumbling table (Mini-Rotator, Thermo Fisher Scientific Inc., Waltham, USA) at room temperature for 30 min. Subsequently, the solution was centrifuged at 1500 rpm at 4 °C. The supernatants were lyophilized and, afterwards, dissolved in PBS (Sigma-Aldrich GmbH, Taufkirchen; Germany). The PBS volume corresponded to the mass of the applied berry skins. The test solution was then sterilized at 70 °C and stored at −20 °C.

### Bacterial species

The following bacterial strains were used in the study: *Fusobacterium nucleatum* (F.n., ATCC 20482), *Aggregatibacter actinomycetemcomitans* (A.a, ATCC 8324), *Porphyromonas gingivalis* (P.g, ATCC 20709), *Enterococcus faecalis* (E.f., ATCC 20376), *Streptococcus mutans* (S.m., ATCC 20523), *Streptococcus sobrinus* (S.s., ATCC 20742), *Streptococcus gordonii* (S.g., ATCC 6777), *Actinomyces naeslundii* (A.n., ATCC 17233).

S.m., S.g., and S.s. were grown in tryptose soy medium (Merck KGaA, Darmstadt, Germany). F.n., P.g., A.a., A.n., and E.f. were cultured in Schaedler’s medium (Carl Roth GmbH, Karlsruhe, Germany) supplemented with vitamin K (10 mg/L). All bacterial strains were grown in 10 ml of liquid medium under anaerobic standard conditions (10% CO_2_, 10% H_2_, 80% N_2_) at 37 °C for 16 to 20 h. Subsequently, bacterial cultures were centrifuged at 4000 rpm for 5 min and the supernatants were removed. The bacterial pellets were washed twice with PBS and then re-suspended in PBS to an optical density of OD_546nm_ = 0.5 (corresponds to 10^6^ CFU/ml).

### Direct contact test

10 µl of the respective bacterial suspensions were gently mixed with 90 µl of each tested solution (AFJ, SJ, NDFM, AFBS, AQFBS) and incubated for 60 s at 37 °C. Subsequently, 900 µl of PBS was added to each tested mixture. The resulting solutions were centrifuged at 4000 rpm for 5 min. The bacterial pellets were dissolved in 200 µl PBS and further diluted in 10-fold steps up to 10^− 5^. 20 µl of each original solution and from the dilution series were plated onto 6% Schaedler blood agar or TS agar plates in accordance with the strain-specific culture conditions, followed by anaerobic incubation for 3–5 days at 37 °C. Afterwards, the number of colony-forming units (CFU/ml) for each solution was determined. 0.2% chlorhexidine (CHX, Chlorhexamed, Haleon, Weybridge, Surrey, UK) served as a positive control and PBS as a negative control. For each test solution, *n* = 8 samples were prepared. Experiments were run in duplicates.

### Statistical analysis

Significant differences were analyzed using the non-parametric Mann-Whitney U-Test. For statistical analysis, the software SPSS version 29.0 was used. The statistical significance was specified at *p* < 0.05, with the values being subsequently adjusted in accordance with the Holm method.

## Results

**Fig. 1:**
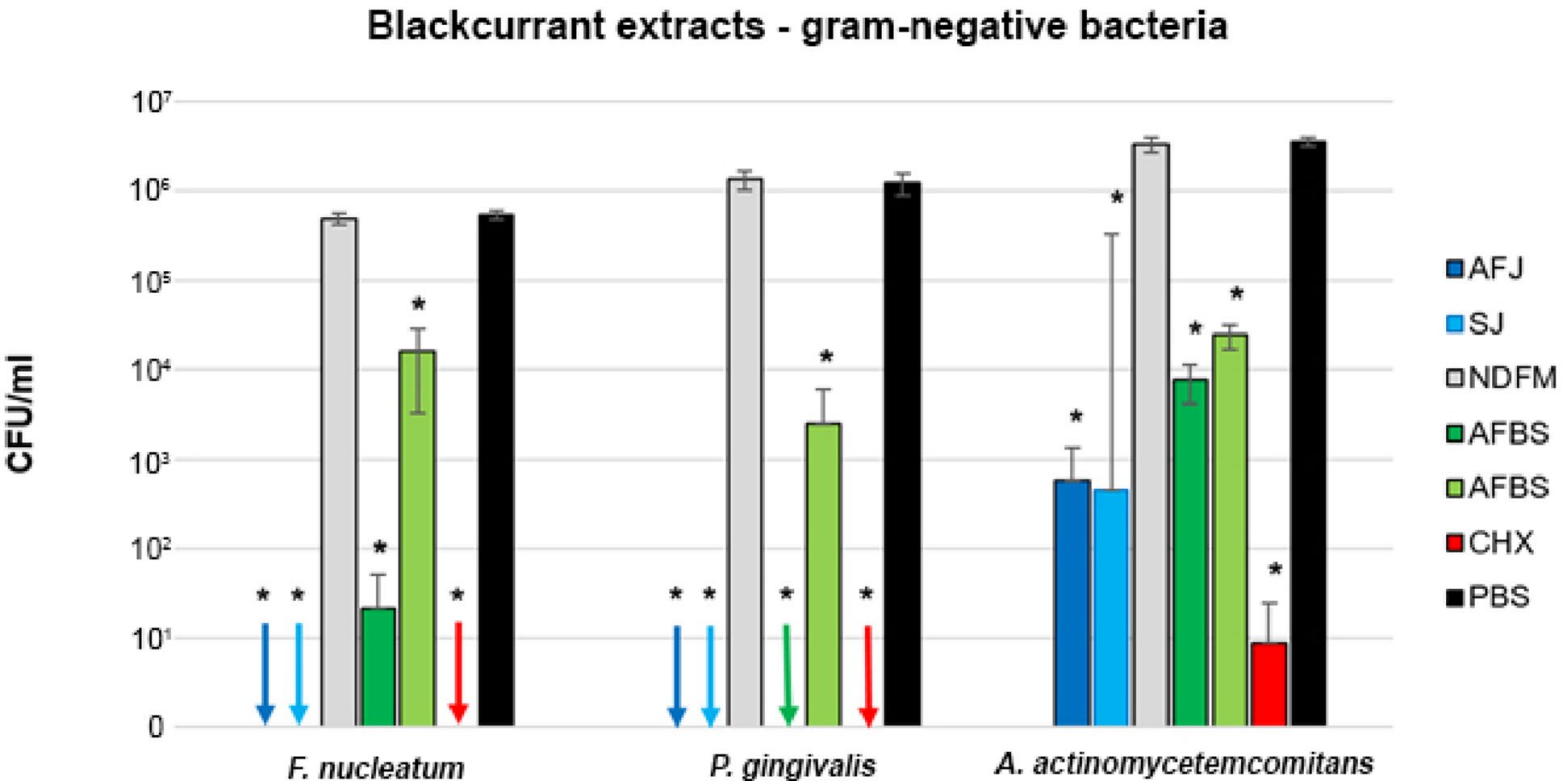
Antibacterial effect of diverse blackcurrant extracts on gram-negative oral bacteria. A significant difference to the negative control (PBS) is marked by a star (*p* < 0.05). Arrows represent complete suppression. (AFJ: all-fruit juice; SJ: squeezed juice; NDFM: non-dialyzable fruit material; AFBS: alcoholic fraction from berry skins; AQFBS: aqueous fraction from berry skins; CHX: chlorhexidine 0.2%; PBS: phosphate buffered saline)

**Fig. 2:**
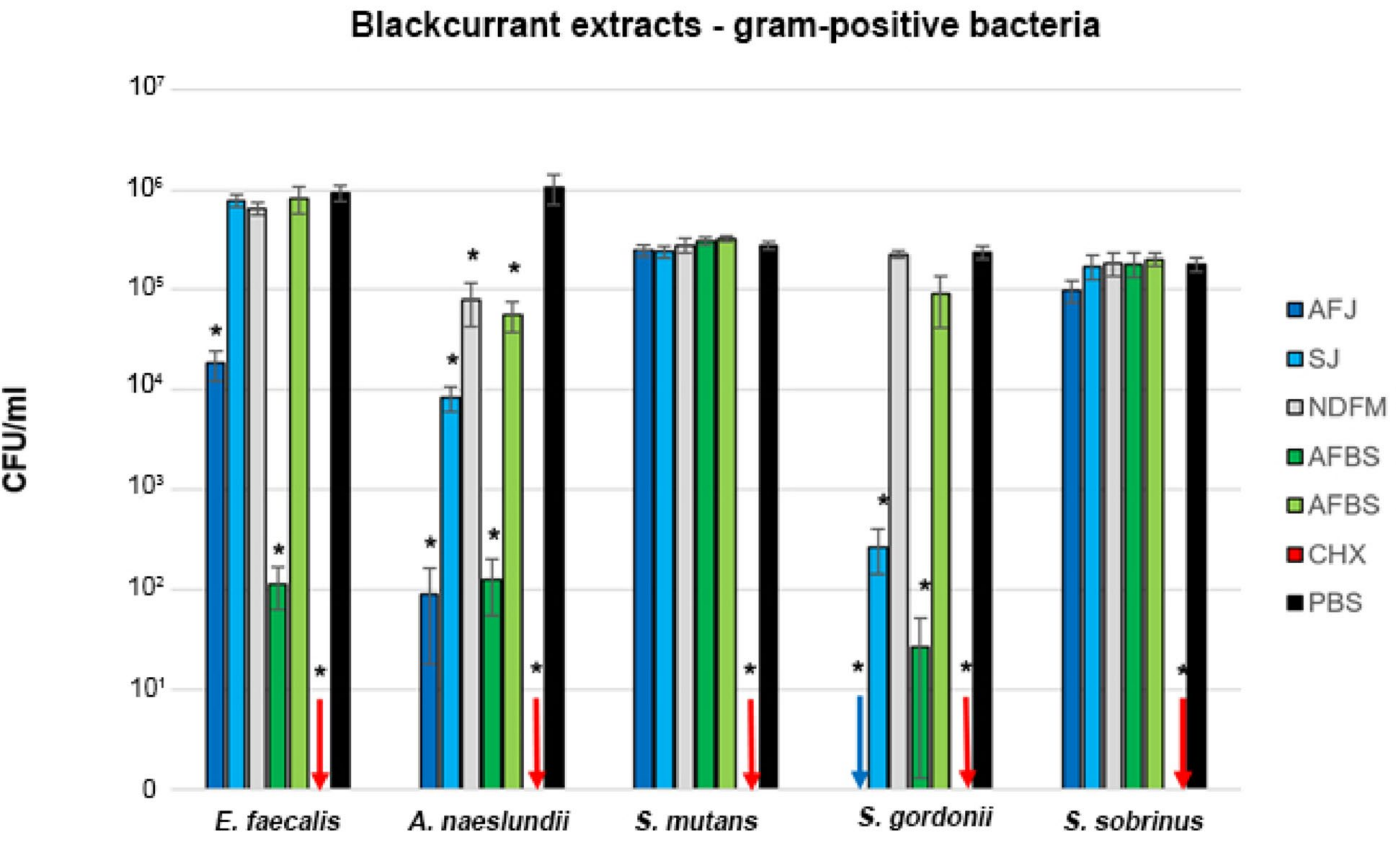
Antibacterial effect of diverse blackcurrant extracts on gram-positive oral bacteria. A significant difference to the negative control (PBS) is marked by a star (*p* < 0.05). Arrows represent complete suppression. (AFJ: all-fruit juice; SJ: squeezed juice; NDFM: non-dialyzable fruit material; AFBS: alcoholic fraction from berry skins; AQFBS: aqueous fraction from berry skins; CHX: chlorhexidine 0.2%; PBS: phosphate buffered saline)

In the direct contact test, the antibacterial efficiency of blackcurrant juices depended on the bacterial species and the type of blackcurrant extract applied (Figs. 1 and 2). In detail, from all tested solutions, AFJ presented the highest antibacterial activity, followed by AFBS > SJ > AQFBS > NDFM. In general, gram-negative bacteria were more susceptible than gram-positive species (Table 1).

**Table 1:** Bacterial reduction in log_10_ CFU/ml compared to the negative control (PBS) for gram-negative oral bacteria

Gram-negative bacteria						
	*F. nucleatum*		*P. gingivalis*		*A. actinomycetemcomitans*	
AFJ	− 5.54	*p* < 0.001	− 6.12	*p* < 0.001	− 3.78	*p* < 0.001
SJ	− 5.54	*p* < 0.001	− 6.12	*p* < 0.001	− 3.91	*p* < 0.001
NDFM	− 0.05	*p* = 0.226	− 0.01	*p* = 0.563	− 0.02	*p* = 0.671
AFBS	− 4.33	*p* < 0.001	− 6.12	*p* < 0.001	− 2.58	*p* < 0.001
AQFBS	− 1.38	*p* < 0.001	− 2.88	*p* < 0.001	− 2.11	*p* < 0.001
CHX	− 5.54	*p* < 0.001	− 6.12	*p* < 0.001	− 5.48	*p* < 0.001

Significance was determined by Mann-Whitney U-Test (p<0.05) compared to the control (PBS). (AFJ: all-fruit juice; SJ: squeezed juice; NDFM: non-dialyzable fruit material; AFBS: alcoholic fraction from berry skins; AQFBS: aqueous fraction from berry skins; CHX: chlorhexidine 0.2%; PBS: phosphate buffered saline)

As shown by the results, incubation with AFJ caused complete suppression of *F. nucleatum*, *P. gingivalis* (Fig. 1, dark blue arrows), and *S. gordonii* (Fig. 2, dark blue arrow). High inhibitory effects of AFJ were also observed for *A. actinomycetemcomitans* (Fig. 1) and A. *naeslundii* (Fig. 2). On all other tested bacteria, AFJ presented only minor suppressive effects.

In Fig. 2, the results for all tested gram-positive species are shown. While *E. faecalis* was still reduced by 1.8 log_10_ in CFU/ml (*p* < 0.05, Table 2), no significant antibacterial activity of AFJ was observed for *S. mutans* and *S. sobrinus*.

SJ and AFBS also showed strong antibacterial activity. Incubation with SJ caused complete suppression of *F. nucleatum* and *P. gingivalis* (light green arrows, Fig. 1), while *P. gingivalis* growth was also completely inhibited by AFBS (Fig. 1, dark green arrow). Furthermore, AFBS induced a major reduction in CFU/ml for *F. nucleatum*,* A. actinomycetemcomitans*,* E. faecalis*,* S. gordonii*, and *A. naeslundi*i (Figs. 1 and 2).

As with AFJ, both SJ and AFBS failed to significantly inhibit the growth of *S. mutans* and *S. sobrinus* (Fig. 2; Table 2).

When compared to the negative control (PBS), aqueous extracts obtained from berry skins (AQFBS) induced minor, but still significant, suppressive effects on *F. nucleatum*, *A. actinomycetemcomitans*,* P. gingivalis*,* S. gordonii*, and *A. naeslundii*. There was no significant inhibitory effect of AQFBS on *E. faecalis*,* S. mutans*, and *S. sobrinus* (Fig. 2; Table 2).

From all tested blackcurrant fractions, NDFM showed the lowest antibacterial activity. Significant suppression was only detected for *A. naeslundii* (Fig. 2; Table 2). In contrast, treatment with 0.2% CHX (positive control) caused complete suppression of all planktonic strains, except *A. actinomycetemcomitans* (red column, Fig. 1). In the case of *F. nucleatum*,* P. gingivalis*, and *S. gordonii*, blackcurrant AFJ showed identical inhibitory activity as 0.2% CHX, resulting in complete suppression of these species (Figs. 1 and 2, dark blue arrows).

**Table 2:** Bacterial reduction in log_10_ CFU/ml compared to the negative control (PBS) for gram-positive oral bacteria

Gram-positive bacteria										
	*E. faecalis*		*A. naeslundii*		*S. mutans*		*S. gordonii*		*S. sobrinus*	
AFJ	− 1.77	*p* < 0.001	− 4.21	*p* < 0.001	− 0.03	*p* = 0.153	− 5.24	*p* < 0.001	− 0.20	*p* = 0.001
SJ	− 0.16	*p* = 0.141	− 2.27	*p* < 0.001	− 0.03	*p* = 0.057	− 2.97	*p* < 0.001	− 0.01	*p* = 0.596
NDFM	− 0.30	*p* = 0.053	− 1.31	*p* < 0.001	0.00	*p* = 0.752	− 0.01	*p* = 0.634	0.00	*p* = 0.634
AFBS	− 3.84	*p* < 0.001	− 3.98	*p* < 0.001	− 0.30	*p* = 0.111	− 3.98	*p* < 0.001	0.00	*p* = 0.832
AQFBS	− 0.12	*p* = 0.528	− 1.54	*p* < 0.001	− 0.05	*p* = 0.417	− 0.34	*p* = 0.832	− 0.02	*p* = 0.064
CHX	− 5.95	*p* < 0.053	− 6.11	*p* < 0.001	− 5.28	*p* < 0.001	− 5.24	*p* < 0.001	− 5.18	*p* < 0.057

Significance was determined by Mann-Whitney U-Test (*p* < 0.05) to the control (PBS). (AFJ: all-fruit juice; SJ: squeezed juice; NDFM: non-dialyzable fruit material, AFBS: alcoholic fraction from berry skins; AQFBS: aqueous fraction from berry skins; CHX: chlorhexidine 0.2%; PBS: phosphate buffered saline)

## Discussion

In the present investigation, diverse fractions from blackcurrant fruits were tested for their efficiency to suppress oral pathogenic bacteria associated with periodontitis, caries, and root canal infection.

Five different types of fruit aliquots were prepared and tested in direct contact with the bacteria. As shown by the results, the antibacterial efficiency differed strongly among the single fruit fractions and depended on the respective bacterial species. It was found that all-fruit juice (AFJ) was the most efficient, followed by the antibacterial activity of the alcoholic fraction from berry skins (AFBS) and squeezed juice (SJ). Aliquots from aqueous fractions of berry skins (AQFBS) and non-dialyzable fruit material (NDFM) showed only minor effects. Therefore, they present no H0 potential.

As shown by the results, direct incubation with AFJ for 60 s completely inhibited the growth of *F. nucleatum*,* P. gingivalis*, and *S. gordonii*. In general, gram-negative bacteria were more susceptible compared to the examined gram-positive species. The high antibacterial activity, especially of AFJ and SJ against *F. nucleatum* and *P. gingivalis*, is in line with previously published data from our research group [6].

Berry phenolics (such as anthocyanins, ellagitannins, and phenolic acids) interact with bacteria via cell membrane disruption, enzyme inhibition, and iron chelation [31, 32]. Generally, gram-positive bacteria are more sensitive to these compounds compared to gram-negative bacteria, which are protected by an additional outer membrane rich in lipopolysaccharides (LPS), acting as a robust barrier against hydrophobic phenolic compounds. Consequently, they display much higher resistance [31–34].

The results of the present study contradict the available data. The following section outlines several speculative assumptions that could plausibly explain this behavior. However, given the lack of experimental substantiation, they should clearly be viewed as hypotheses rather than definitive explanations.

The higher susceptibility of gram-negative species in the present investigation might be caused by the chelating activity of anthocyanins like delphinidin-3-rutinoside and cyanidin-3-rutinoside that are present in blackcurrant fruits in high concentrations. In gram-negative bacteria, chelation of Mg^2+^ and Ca^2+^ in the outer membrane may induce its destabilization, followed by a severe loss of membrane integrity [35, 36]. Additionally, blackcurrant fruit juice and extracts natively possess a low pH due to a very high concentration of weak organic acids, predominantly citric acid. In its un-ionized, lipophilic form, citric acid passes through the gram-negative outer membrane via passive diffusion or through porin protein channels, causing a pH drop in the bacterial interior. Because gram-positive bacteria have a thick peptidoglycan cell wall that acts as a buffer against rapid charge shifts, they can sometimes tolerate these sudden acidic microenvironments slightly better than the thin-walled gram-negative species [37]. Furthermore, the primary active compounds in blackcurrants are small, low-molecular-weight monomeric anthocyanins and phenolic acids (like caffeic and p-coumaric acids), which can easily diffuse through the outer membrane porins of gram-negative bacteria to access the vulnerable inner cytoplasmic membrane. This might be another reason why gram-negative bacteria were more susceptible compared to gram-positive in the present investigation.

In the present study, an exposure time of 60 s was chosen, which is the maximum recommended contact time for mouthwashes [38–40]. The evaluation of multiple incubation periods would probably have enabled a more comprehensive understanding of the time-dependent antibacterial activity of each blackcurrant fraction and would have reflected a more realistic scenario of oral exposure. This issue can be considered a further limitation of the present investigation.

Overall, studies evaluating the antimicrobial activity of berry fruit extracts on oral pathogens are still rare. Available data predominantly focus on bacterial biofilms, rather than on single planktonic oral species. Therefore, the present study also discusses data obtained from oral mono-species biofilms.

In this regard, a recent study confirmed the inhibitory effect of blackcurrant fruit extracts (AFJ, AFBS) on 1-, 4-, and 24-h old ex vivo biofilms. Chlorhexidine (CHX, 0.2%) served as a positive control. Treatment with AFJ and CHX was more effective in inhibiting biofilm growth compared to AFBS. Early biofilms (1- and 4-h old) were more susceptible to AFJ and CHX compared to 24-h old biofilms. Therefore, the inhibitory activity of AFJ was similar to that of 0.2% CHX [41].

Besides blackcurrant juices, especially blueberry extracts show strong antibacterial activity against *F. nucleatum* [42]. This property may result from the ability of blueberry polyphenols to chelate iron. Moreover, it was found that blueberry extracts (62.5 µg/ml) are capable of inhibiting *F. nucleatum* biofilm formation by 87.5 ± 2.3% [42]. Whether blackcurrant extracts also inhibit *F. nucleatum* biofilm formation still needs to be discovered.

In the present study, *P. gingivalis* was also highly susceptible to blackcurrant juice. In particular, AFJ, SJ and AFBS completely inhibited its growth. This is in line with investigations that report the capability of proanthocyanidins to suppress *P. gingivalis* [43]. It was proven that especially proanthocyanidins, such as procyanidin B2 and epigallocatechin can react with gingipains and hemagglutinin, thus influencing the adhesion ability and virulence of *P. gingivalis* [44, 45]. In this regard, the reason blackcurrants exhibit antibacterial activity against *P. gingivalis* could be that they possess high levels of polyphenols, specifically anthocyanins and proanthocyanidins [21].

Antibacterial activity of blackcurrant AFJ was also observed in the present study for *A. actinomycetemcomitans* and *A. naeslundii*. While there were still some bactericidal effects on *E. faecalis* by AFBS, none of the prepared juice fractions showed any prominent inhibitory activity on the gram-positive species *S. mutans* and *S. sobrinus*.

In the present study, *S. gordonii* was the only gram-positive species completely suppressed by blackcurrant extracts. Application of AFJ entirely restricted its growth. This is in line with the former observations of our group, which confirmed the strong suppressive activity of blackcurrant juice (concentration 90%, incubation time 60 s) on *S. gordonii* [6].

In accordance with the present investigation, other authors also reported the inability of blackcurrant fruit extracts to suppress gram-positive *S. mutans* [46]. It was observed that blackcurrant juice in concentrations < 20% is not capable of inhibiting this species. Detailed analysis verified that ferulic acid and chlorogenic acid seem to be the main substances in blackcurrant juice extracts that have a suppressive effect on *S. mutans* [29].

In general, oral streptococci are known to be early colonizers of the enamel and to co-aggregate with other pioneer species, such as certain Actinomyces spp. In another study, the effect of blackcurrant juice on inhibiting the formation of bacterial aggregates was observed. It was found that blackcurrant juice is able to prevent and to reverse bacterial co-aggregation of *S. mutans* with *A. naeslundii* [47]. These effects were attributed to the polyphenols that are present in blackcurrant fruit juices in concentrations from 0.6 to 1.4 mg/g [47]. Furthermore, it was found that proanthocyanidins such as (epi)catechin or (epi)gallocatechin can bind to pellicle proteins as well as to α-amylase and thus compete with pioneer species for enamel adhesion [48]. In this context, it was also discovered that the presence of cranberry polyphenols, especially flavonols, can impair the activity of bacterial glucosyl- and fructosyltransferases, causing a loss in bacterial enamel adhesion and biofilm formation capability [49].

In the present study, the obtained fruit juices were heated to 70 °C for sterilization. Heat treatment might negatively affect the stability and bioactivity of anthocyanins and polyphenols. It was found that temperatures of 70 °C for 1 to 2 h can cause a drop in bioactivity up to 20% [50].

A further limitation of the study is the lack of compositional analysis. A detailed characterization and quantification of the active components would have been of advantage. Therefore, the following paragraph reviews the main active substances that are present in blackcurrant fruit juice and pomace.

Previous analyses showed that blackcurrants possess high amounts of anthocyanins (97%). These include delphinidin-3-*O*-rutinoside, cyanidin-3-*O*-rutinoside, delphinidin-3-*O*-glucoside and cyanidin-3-*O*-glucoside. Besides anthocyanins, flavonols (quercetin, myricetin, kaempferol derivatives, isorhamnetin glycosides), phenolic acids (hydroxycinnamic and hydroxybenzoic acids), flavanols and proanthocyanidins (catechin, epicatechin) were also present [51, 52].

The ethanolic fraction is mainly composed of anthocyanins and, to a lesser extent, also of flavonols, flavonol glycosides, flavanols, proanthocyanidins (tannins), flavanonols (dihydroflavonols), and phenolic and organic acids.

The aqueous fraction mainly includes water-soluble pigments (anthocyanins), but also hydrophilic flavonol glycosides (rutin, isoquercetin), soluble flavanols, condensed tannins, organic fruit acids, and free sugars (d-glucose, d-fructose), as well as essential minerals (K, P, Mg, Ca) [22, 53].

In the present investigation, incubation with AFJ and SJ caused only minor effects on *E. faecalis*. From all fruit fractions prepared, AFBS still showed the strongest antibacterial activity. Direct contact with AFBS caused a decrease in *E. faecalis* by 3.8 log CFU/ml. Up to now, there are no further studies available that report a bactericidal effect of blackcurrant extracts on *E. faecalis*. Apparently, the species reacts differently to plant phenols from diverse plant extracts. While extracts from wild rose, chokeberry, and elderberry showed no antibacterial effects, fractions from cranberry, Japanese quince, and sea buckthorn caused strong growth inhibition of *E. faecalis* [28].

In summary, from all tested extracts, AFJ was the most efficient. Incubation with AFJ caused complete suppression of *F. nucleatum*, *P. gingivalis*, and *S. gordonii* and induced a major reduction of A.a., A.n., and E.f. In contrast, *S. mutans* and *S. sobrinus* were the most resistant against blackcurrant extracts. In both cases, only treatment with 0.2% CHX caused their total inhibition.

## Conclusions

In the present study, it was verified that AFJ from blackcurrant fruits has an antibacterial effect on certain gram-negative and gram-positive oral pathogenic bacteria. Efficient antibacterial activity was also proven for alcoholic extracts derived from berry skins, which are significantly rich in polyphenols. Further studies are necessary for evaluating the capability of berry phenols to control oral plaque bacteria. In future investigations, our research group will also focus on specific drug carrier systems that might enable a more effective interaction of such natural compounds with bacterial cell walls.

## Data Availability

Data can be obtained on request from the corresponding author.

## Abbreviations

A.a.: *Aggregatibacter actinomycetemcomitans*
A.n.: *Actinomyces naeslundii*
AFBS: Alcoholic fraction from berry skins
AFJ: All-fruit juice
AQFBS: Aqueous fraction from berry skins
CFU: Colony-forming units
CHX: Chlorhexidine
DMSO: Dimethyl sulfoxide
E.f.: *Enterococcus faecalis*
F.n.: *Fusobacterium nucleatum*
MW: Molecular weight
MWC: Molecular weight cut-off
NDFM: Non-dialyzable juice material
P.g.: *Porphyromonas gingivalis*
PBS: Phosphate buffered saline
S.g.: *Streptococcus gordonii*
S.m.: *Streptococcus mutans*
S.s.: *Streptococcus sobrinus*
SJ: Squeezed juice
